# Hearing Recovery Prediction for Patients with Chronic Otitis Media Who Underwent Canal-Wall-Down Mastoidectomy

**DOI:** 10.3390/jcm13061557

**Published:** 2024-03-08

**Authors:** Minsu Chae, Heesoo Yoon, Hwamin Lee, June Choi

**Affiliations:** 1 Department of Biomedical Informatics, Korea University College of Medicine, Seoul 02841, Republic of Korea; minsuchae@korea.ac.kr; 2 Department of Otorhinolaryngology-Head, Head and Neck Surgery, Korea University Ansan Hospital, Ansan-si 15355, Republic of Korea; yhsjoa@hanmail.net

**Keywords:** chronic otitis media, hearing recovery, machine learning, canal-wall-down mastoidectomy

## Abstract

**Background:** Chronic otitis media affects approximately 2% of the global population, causing significant hearing loss and diminishing the quality of life. However, there is a lack of studies focusing on outcome prediction for otitis media patients undergoing canal-wall-down mastoidectomy. **Methods:** This study proposes a recovery prediction model for chronic otitis media patients undergoing canal-wall-down mastoidectomy, utilizing data from 298 patients treated at Korea University Ansan Hospital between March 2007 and August 2020. Various machine learning techniques, including logistic regression, decision tree, random forest, support vector machine (SVM), extreme gradient boosting (XGBoost), and light gradient boosting machine (light GBM), were employed. **Results:** The light GBM model achieved a predictive value (PPV) of 0.6945, the decision tree algorithm showed a sensitivity of 0.7574 and an F1 score of 0.6751, and the light GBM algorithm demonstrated the highest AUC-ROC values of 0.7749 for each model. XGBoost had the most efficient PR-AUC curve, with a value of 0.7196. **Conclusions:** This study presents the first predictive model for chronic otitis media patients undergoing canal-wall-down mastoidectomy. The findings underscore the potential of machine learning techniques in predicting hearing recovery outcomes in this population, offering valuable insights for personalized treatment strategies and improving patient care.

## 1. Introduction

Chronic otitis media (COM) is a medical condition characterized by persistent inflammation of the middle ear lasting over three months. The condition may result from an inability to maintain proper air pressure in the middle ear or from ear infections that lead to a perforated eardrum [1]. The main symptoms commonly associated with COM include ear discharge, hearing loss, tinnitus, vertigo, facial nerve palsy, and otalgia. Moreover, COM-associated inflammation can result in the erosion of the ossicles in the middle ear and the potential dissemination of infection to the brain. Furthermore, the aforementioned condition can also cause hearing loss, thus hindering efficient communication and reducing the overall quality of life [2,3]. Data indicate that COM is a prevalent condition, affecting approximately 2% of the global population, and has been linked to a decreased quality of life due to hearing loss [4]. To improve overall well-being, prioritizing the treatment of COM and the recovery of hearing function is crucial [5].

The treatment of COM involves both pharmaceutical and surgical interventions. In mild cases, pharmaceutical treatments typically involve antibiotics and dietary modifications [6]. Surgical treatments include canal-wall-down mastoidectomy (CWD) and tympanoplasty surgeries, with CWD being the most commonly used approach [7]. Additionally, CWD offers improved surgical visualization and reduces recurrence rates [8]. However, recognizing that not all patients undergoing CWD experience hearing recovery is necessary because outcomes can vary among individuals [5]. The objective of this study was to use machine learning to predict the hearing restoration prognoses in patients with COM undergoing CWD treatments. The study encompasses a broader spectrum of COM, including conditions such as tympanosclerosis, middle ear cholesteatoma, and other related pathologies, beyond simple perforation of the tympanic membrane. This is expected to play a significant role in prognostic prediction and contribute to hearing recovery under various conditions.

## 2. Materials and Methods

### 2.1. Data Collection

Data were collected from 321 patients diagnosed with COM who underwent CWD surgery at Korea University Ansan Hospital between March 2007 and August 2020. CWD mastoidectomy, as described in our surgical approach, traditionally involves both the reconstruction of the posterior wall and mastoid obliteration, considered integral components of the procedure. However, it is important to note that, in our surgical practice, mastoid obliteration was performed for all patients as part of the procedure, aligning with the aim of filling the mastoid cavity with autologous materials to prevent retraction pocket formation and the subsequent recurrence of disease. Conversely, the reconstruction of the posterior wall was not uniformly undertaken in all cases. This decision was made based on a thorough assessment of individual patient factors and surgical considerations. While posterior wall reconstruction aims to restore the anatomical integrity of the middle ear and provide structural support, its omission in certain cases was deemed appropriate to minimize surgical complexity and associated risks while still achieving the primary goal of mastoid cavity obliteration.

We selected relevant features, guided by our clinical expertise, and excluded duplicate or incomplete data. The exclusion criteria were as follows: (1) duplicate samples, (2) patients with missing values for the stapes attribute, and (3) patients with missing values for the tympanoplasty technique. A total of 298 patients with COM underwent surgery. The statistical analysis results for the participants are presented in Table 1. Of these, 126 experienced hearing improvements, whereas 172 did not. We conducted 10-fold cross-validation due to a small dataset size of 298 patients. During each cross-validation iteration, an algorithm was trained on data from 268 patients and evaluated using data from 30 patients. We categorized the tympanoplasty technique as applying overlay. From the patient cohort, 254 individuals exhibited evidence of cholesteatoma, while tympanosclerosis was identified in 66 patients. Additionally, tympanic membrane perforation was observed in 123 cases.

### 2.2. Definition of Recovery

Recovery from COM was defined based on the pure-tone average (PTA) test, which includes the parameters in Table 2. The PTA test was administered at the interval of 6 months postoperation.

We considered hearing recovery to have occurred if any of the following criteria were met: (1) postoperative AC PTA was ≤30 dB; (2) postoperative ABG was ≤20 dB; or (3) the difference between preoperative AC PTA and postoperative AC PTA was ≥15 dB.

### 2.3. Machine Learning Models

We employed machine learning techniques to forecast hearing recovery, utilizing various models commonly utilized in medical research. However, deep learning was omitted from consideration due to insufficient data availability. Owing to our limited dataset, we validated these models using a cross-validation method.

(1) Logistic Regression

This statistical method is used to classify outcomes based on regression results. This process involves calculating the sigmoid function by considering each attribute and weight-related attribute. The results of the sigmoid function are then determined. If the result is ≥0.5, the patient is predicted to recover. Otherwise, hearing loss is not predicted to subside [9].

(2) Decision Tree

This involves the creation of nodes that enable the classification of hearing recovery based on attributes and thresholds [10]. Samples are used for determination. To construct a rule, we determined the feature that maximized the impurity and calculated its corresponding threshold.

(3) Random Forest

This technique, called ensemble modeling, involves constructing several decision trees and aggregating their predictions [11]. Random forest uses bootstrap aggregation to apply different attributes to multiple subsamples and each subsample individually. Moreover, the random forest algorithm uses the combined outcomes of several decision trees to predict hearing recovery.

(4) Support Vector Machine (SVM)

This technique obtains samples located around decision boundaries and aims to increase the distances between them [12,13]. The SVM enables the effective prediction of hearing recovery, particularly for unseen data.

(5) Extreme Gradient Boosting (XGBoost)

Gradient boosting is a technique used to minimize the remaining error in the hearing recovery procedure, by repeatedly training several models within a unified one. The extreme gradient boosting variation uses parallelization methods and implements tree pruning [14,15].

(6) Light Gradient Boosting Machine (Light GBM)

The light GBM calculation is derived from a histogram. This approach enhances the functionality of XGBoost and exhibits comparable performance [14,15].

### 2.4. Evaluation Metrics

Machine learning involves the analysis of performance metrics to determine the accuracy of expected outcomes. In this study, we aimed to evaluate and contrast the recuperations of different patients diagnosed with COM. When calculating accuracy in both recovered and non-recovered patients, this parameter is considered unacceptable for use as a performance evaluation metric in medical data. Furthermore, when analyzing medical data, predicting the occurrence of a disease or the likelihood of recovery is crucial. We used the functions provided by the Scikit-learn library.

### 2.5. Feature Selection

In machine learning, the significance of having accurate features surpasses that of having numerous other features. We used a sequential feature selector from the mlxtend library [16] to optimize the feature selection for each model and effectively choose relevant features from the given combinations to enhance the model’s performance. Generating all possible feature combinations to determine the optimal set can be challenging. The sequential feature selector method allows us to effectively select relevant features from a series of feature combinations provided, thereby identifying suitable features based on their characteristics [17].

## 3. Results

### 3.1. Feature Screening Results

**Figure 1:**
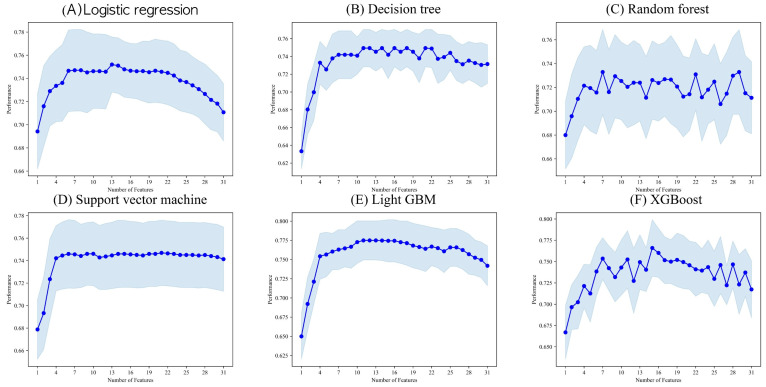
Results of performing the sequential feature selector algorithm.

Figure 1 illustrates the correlation between the algorithm’s performance and the number of attributes, using the area under the receiver operating characteristic curve for comparison. The selected patient characteristics for each algorithm were as follows: (1) logistic regression: sex, age, recurrence, hypertension, smoking history, retraction, presence of cholesteatoma, intraoperative eustachian tube findings, facial nerve canal, malleus, incus, ossicular status, total score, preoperative AC PTA, preoperative BC PTA, preoperative ABG, and CWD surgery characteristics; (2) decision tree: sex, age, diabetes mellitus, hypertension, intraoperative eustachian tube findings, tympanoplasty technique, malleus, incus, preoperative AC PTA, preoperative BC PTA, and preoperative ABG characteristics; (3) random forest: sex, age, intraoperative eustachian tube findings, stapes fixation, preoperative AC PTA, preoperative ABG, and CWD surgery characteristics; (4) SVM: sex, age, recurrence, diabetes mellitus, smoking pack-years, tympanic membrane condition, perforation margin tympanosclerotic plaque (TSP), attic destruction, preoperative otorrhea, preoperative culture, tympanoplasty technique, malleus, incus, stapes fixation, ossicular quality, middle ear, previous surgeries, preoperative BC PTA, preoperative ABG, CWD surgery, and intact bridge mastoidectomy (IBM) surgery characteristics; (5) light GBM: age, recurrence, smoking history, smoking pack-years, retraction, intraoperative eustachian tube findings, intraoperative culture, tympanoplasty technique, malleus, preoperative BC PTA, preoperative ABG, and CWD surgery characteristics; (6) XGBoost: age, recurrence, retraction, attic destruction, preoperative otorrhea, the presence of cholesteatoma, intraoperative eustachian tube findings, facial nerve canal, middle ear, previous surgery, preoperative BC PTA, preoperative ABG, and CWD surgery characteristics.

The common attribute used across all of the algorithms was age. Five models were utilized with preoperative ABG, preoperative BC PTA, and intraoperative eustachian tube insights as characteristics. Age and preoperative ABG were considered important attributes in all of the models. The essential attributes included intraoperative eustachian tube findings and preoperative BC PTA, which were used in all five models.

### 3.2. Performance Results

Performance evaluation was rigorously conducted through cross-validation, with the results for each algorithm methodically presented in Table 3. The performance metrics are as follows: Logistic regression achieved a positive predictive value (PPV) of 0.6322, a sensitivity of 0.6917, and an F1 score of 0.6528. The decision tree algorithm showed a PPV of 0.6218, a sensitivity of 0.7574, and an F1 score of 0.6751. For the random forest model, the PPV was 0.6322, the sensitivity was 0.6917, and the F1 score was 0.6528. The SVM recorded a PPV of 0.6238, a sensitivity of 0.5788, and an F1 score of 0.5917. Light GBM demonstrated the highest PPV among the models at 0.6945, with a sensitivity of 0.5788 and an F1 score of 0.6204. Finally, XGBoost achieved a PPV of 0.6375, a sensitivity of 0.5397, and an F1 score of 0.5777. According to Table 3, which details the machine learning performance metrics, light GBM exhibited the highest PPV, while the decision tree algorithm showed the highest sensitivity and F1 score. The order of performance based on PPV is as follows: light GBM, XGBoost, logistic regression, SVM, decision tree, and random forest. The order of performance based on sensitivity is as follows: decision tree, logistic regression, SVM, light GBM, random forest, and XGBoost. Overall, the decision tree had the best performance. We considered both PPV and sensitivity. If considering only PPV, light GBM showed the best performance.

**Figure 2:**
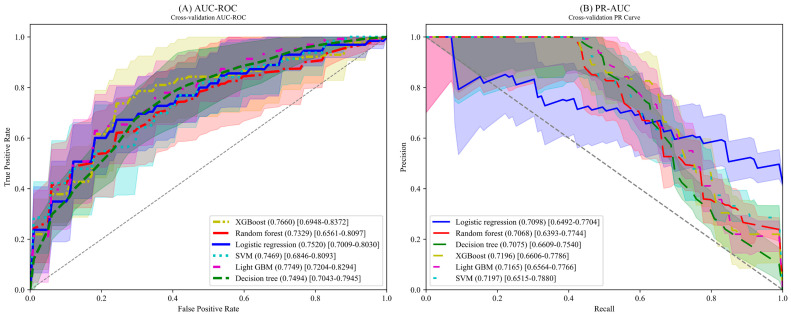
The red color refers to the random forest model. The green color refers to the decision tree. The blue color refers to the logistic regression. The cyan color refers to the SVM. The magenta color refers to the light GBM model. The yellow color refers to XGBoost: (**A**) The results of the area under the receiver operating characteristic curve for the various machine learning models are shown. The light GBM models displayed the highest performance. (**B**) The results for the precision–recall curve for the various machine learning models are shown. The XGBoost model displayed the highest performance.

Figure 2A illustrates the area under the receiver operating characteristic (AUC-ROC) for each model, emphasizing that light GBM and XGBoost are the most effective algorithms for this metric. Figure 2B presents the precision–recall curve for each model, with XGBoost emerging as the most efficient algorithm. The AUC-ROC performance metrics were as follows: light GBM at 0.7749, XGBoost at 0.7749, decision tree at 0.7494, logistic regression at 0.7497, SVM at 0.7469, and random forest at 0.7329. Regarding the precision–recall area under the curve (PR-AUC) performance metrics, XGBoost led with 0.7270, followed by SVM at 0.7197, light GBM at 0.7165, the decision tree model at 0.7075, the random forest model at 0.7068, and logistic regression at 0.7024. Based on the F1 score, the decision tree model was ultimately identified as the optimal choice.

Table 4 presents the performance metrics as determined by the false-positive rate (FPR). When the FPR was set at 10%, the analysis showed that the random forest model displayed the highest performance. At an FPR of 20%, the light GBM model exhibited superior performance. Furthermore, when the FPR ranged between 30% and 40%, the decision tree model outperformed the other models under these conditions. Although performance measurements are commonly obtained using a default FPR value of 50%, our findings suggest that adjusting the FPR to 40% results in the most optimal performance.

The logistic regression offered the optimal trade-off based on the threshold compared to other algorithms. The decision tree model had the best performance with sensitivity consideration. The logistic regression exhibited a reduced trade-off based on the threshold, although it did not have the highest PPV. The random forest model appeared to have a large trade-off, depending on the threshold. However, the random forest model displayed the best PPV. Overall, the decision tree had the best performance.

### 3.3. Analysis Results

**Figure 3:**
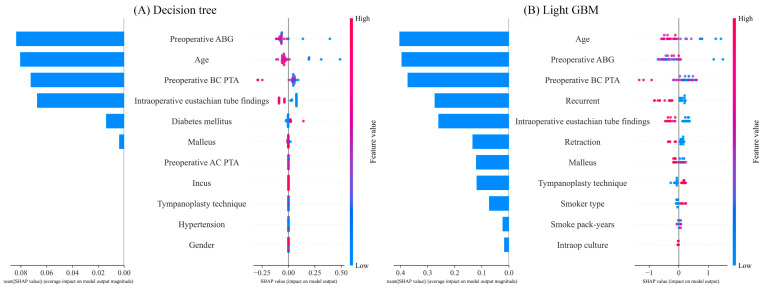
Shapley additive explanation analyses of the two best-performing machine learning models: (**A**) decision tree; (**B**) light GBM.

Our proposed model demonstrated exceptional performance, with a PPV of 0.6218 and a sensitivity of 0.7574. To provide detailed insights, we also conducted Shapley additive explanation (SHAP) analyses on both the decision tree model—which exhibited the highest F1 score—and the light GBM model—which had the highest AUC-ROC [18]. Figure 3 illustrates the SHAP results for the decision tree and light GBM models. The decision tree model analysis revealed that a low preoperative ABG, young age, reduced BC PTA, and the absence of intraoperative eustachian tube abnormalities were positively associated with an increased likelihood of hearing recovery. The light GBM model analysis revealed that young age, a low preoperative ABG, reduced BC PTA, and no recurrent COM were positively associated with an increased likelihood of hearing recovery.

**Figure 4:**
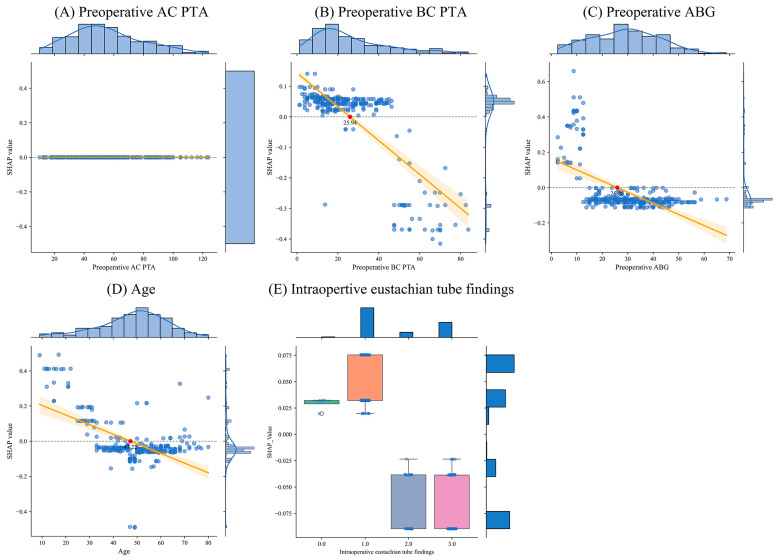
Analysis of distribution and SHAP across various characteristics based on the decision tree model. The orange line represents the mean and standard deviation of linear regression, and the red dot represents the cut-off values.

Figure 4 displays an analysis of the impact on hearing recovery depending on specific features based on the decision tree model. Figure 4A illustrates the analysis results of the SHAP values and preoperative AC PTA. It was confirmed that the preoperative AC PTA was not influenced by improvements in hearing. Figure 4B illustrates the analysis results of the SHAP values and preoperative BC PTA. It was confirmed that a preoperative BC PTA exceeding 42 dB had an adverse impact on hearing recovery. Figure 4C illustrates the analysis results of the SHAP values and preoperative ABG. It was confirmed that preoperative ABG of less than 10 dB contributed to hearing recovery. Figure 4D illustrates the analysis results of the SHAP values and age. It was confirmed that an age of less than 30 years old contributed to hearing recovery. Figure 4E illustrates the analysis results of the SHAP values and the intraoperative eustachian tube. It was confirmed that if the intraoperative eustachian tube was obstructed, there were no improvements in hearing.

**Figure 5:**
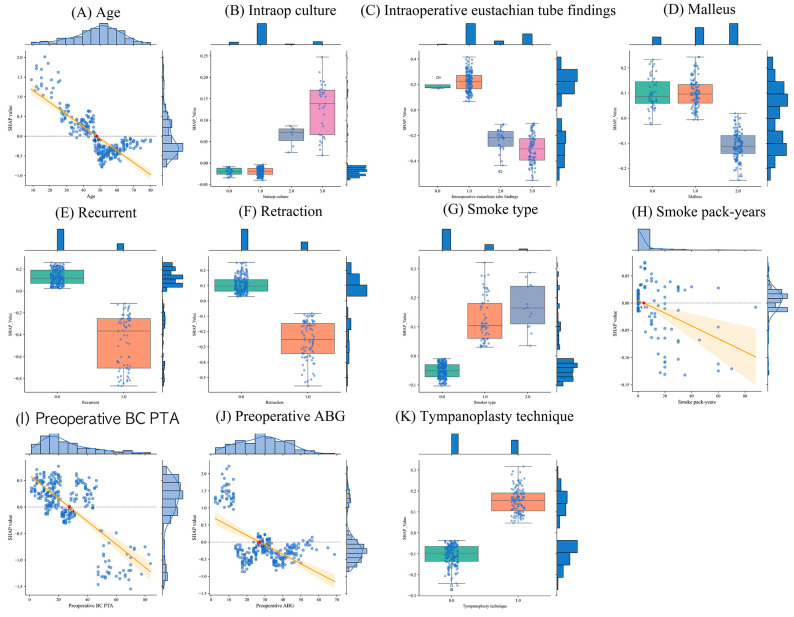
Analysis of distribution and SHAP across various characteristics based on light GBM. The orange line represents the mean and standard deviation of linear regression, and the red dot represents the cut-off values.

Figure 5 displays an analysis of the impact on hearing recovery depending on the specific features based on the light GBM model. Figure 5A illustrates the analysis results of the SHAP values and age. It was confirmed that an age of less than 48 years old contributes to hearing recovery. Figure 5B illustrates the analysis results of the SHAP values and intraoperative culture. It was confirmed that intraoperative culture does not impact hearing recovery. Figure 5C illustrates the analysis results of the SHAP values and the intraoperative eustachian tube. It was confirmed that if the intraoperative eustachian tube was obstructed, there were no improvements in hearing. Figure 5D illustrates the analysis results of the SHAP values and malleus. It was confirmed that if the malleus was removed or defective, there were no improvements in hearing. Figure 5E illustrates the analysis results of the SHAP values and recurrent COM. It was confirmed that if the patient had a history of COM, there were no improvements in hearing. Figure 5F illustrates the analysis results of the SHAP values and retraction. It was confirmed that the retraction of the ear structure impairs hearing recovery. Figure 5G,H illustrates the analysis results of the SHAP values and smoke type and smoke pack-years, respectively. It was confirmed that smoking may seem to improve hearing, but the impact of smoke pack-years is not significant if it is less than 4.3 pack-years. Figure 5I illustrates the analysis results of the SHAP values and preoperative BC PTA. It was confirmed that a preoperative BC PTA exceeding 27 dB has an adverse impact on hearing recovery. Figure 5J illustrates the analysis results of the SHAP values and preoperative ABG. It was confirmed that a preoperative ABG of less than 10 dB contributes to hearing recovery. Figure 5K illustrates the analysis results of the SHAP values and the tympanoplasty technique. It was confirmed that applying the overlay to the tympanoplasty technique improves hearing.

**Figure 6:**
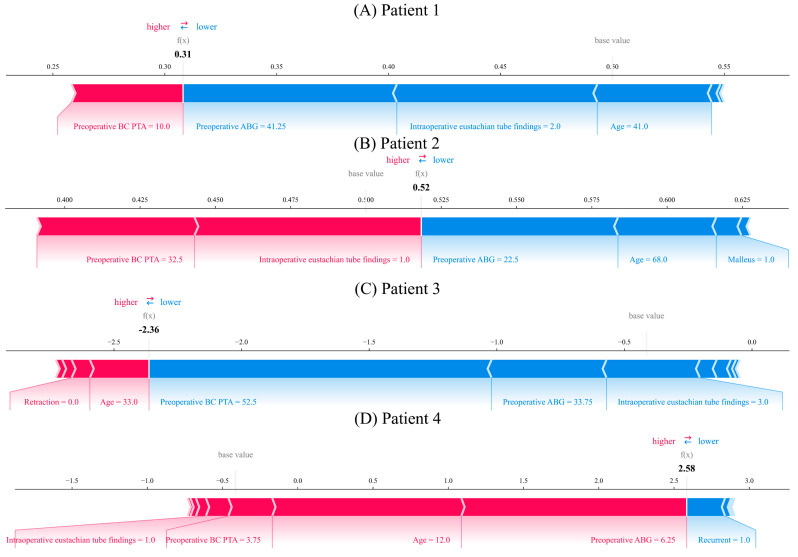
Shapley additive explanation analysis of hearing recovery prediction: (**A**) analysis of patients who did not recover their hearing, based on the decision tree model; (**B**) analysis of patients who recovered their hearing, based on the decision tree model; (**C**) analysis of patients who did not recover their hearing, based on the light GBM; (**D**) analysis of patients who recovered their hearing, based on the light GBM.

Figure 6 provides an extensive SHAP analysis of hearing recovery prediction, furnishing valuable insights into the factors influencing patient outcomes following surgery. Our study involved a comparison between the decision tree and light GBM models through cross-validation, utilizing the last trained model. Additionally, Figure 6 illustrates the selection of a randomly chosen patient with accurately predicted outcomes from the test dataset. Figure 6A focuses on the analytical outcomes for patients who did not recover their hearing, highlighting low preoperative bone-conduction pure-tone average (BC PTA) as a significant contributor to hearing improvement, while a high preoperative air–bone gap (ABG), older age, and Eustachian tube abnormalities during surgery are noted as restrictions. Figure 6B illustrates the results for patients who achieved hearing recovery, emphasizing the importance of a low BC PTA and favorable intraoperative Eustachian tube findings. Conversely, Figure 6C details the factors for patients who did not experience hearing recovery, identifying young age and the absence of retraction as beneficial but also noting the impediments of a high preoperative BC PTA, high preoperative ABG, and Eustachian tube abnormalities during surgery. Lastly, Figure 6D showcases the analytical findings for patients who experienced hearing recovery, analyzed using the light GBM model. Therefore, young age, a low preoperative BC PTA, and ABG were identified as contributing factors to hearing recovery. Obstacles to hearing recovery included a record of recurrence. The results of our analysis exhibited a high correlation with clinical outcomes. The investigation demonstrated that the most significant factors affecting hearing recovery were BC PTA, ABG, and age.

## 4. Discussion

We proposed a recovery prediction model for patients with COM who underwent CWD surgery. Although several studies have aimed to predict hearing recovery in this context, few predictive studies exist on hearing recovery in patients with COM following CWD surgery [19,20,21,22,23]. The decision tree model had the highest performance among the models we presented, achieving a precision of 62.18% and a recall rate of 75.74%. There is a lack of research on predicting hearing recovery in patients with COM who underwent CDW surgery, and therefore it is difficult to compare our results to other papers.

The results obtained from extracting features from our six proposed models were as follows: (1) logistic regression: recurrent, intraoperative eustachian tube findings, age, preoperative BC PTA, retraction; (2) decision tree: preoperative ABG, age, preoperative BC PTA, intraoperative eustachian tube findings; (3) random forest: preoperative ABG, age, preoperative AC PTA, intraoperative eustachian tube findings; (4) SVM: age, preoperative ABG, preoperative BC PTA; (5) light GBM: age, preoperative ABG, preoperative BC PTA, recurrent, intraoperative eustachian tube findings; (6) XGBoost: age, preoperative ABG, recurrent, intraoperative eustachian tube findings. Age, preoperative ABG, and preoperative BC PTA are among the primary factors affecting hearing recovery for patients with COM. Hearing recovery was more probable for patients under 42 years old. Hearing recovery was more probable for preoperative ABG of less than 10 dB. Additionally, if patients have ever had COM, it can have a negative effect on hearing recovery. In other words, it is very important to treat and manage COM to prevent recurrence.

## 5. Conclusions

This study introduced a groundbreaking recovery prediction model designed for patients who have undergone CWD surgery to treat COM. Our machine learning model demonstrated outstanding performance, with a PPV of 0.6218 and a sensitivity of 0.7574. Our study makes a significant impact in the following ways: (1) Our model serves the dual purpose of predicting hearing recovery as well as providing patients with essential information to enhance their overall hospital satisfaction, and (2) medical practitioners may benefit from our model by offering valuable guidelines for hearing recovery that are grounded upon robust evidential support. Age, preoperative BC PTA, and preoperative ABG were identified as the primary factors determining hearing recovery among patients with COM. The decision tree model was the best performance model for predicting hearing recovery in patients with COM. The random forest model exhibited the highest PPV when adjusted based on the FPR threshold. The limitations of our study are as follows: (1) We conducted a single-cohort study and did not perform external validation on our model, and (2) our dataset consisted of a relatively modest sample size, encompassing 298 patients, potentially constraining the model’s generalizability. Our objective was to develop a web-based hearing recovery prediction assistant system for patients with COM and assess the effectiveness of the system among medical professionals.

## Figures and Tables

**Table 1:** Characteristics and statistical analysis of the patients.

General Characteristics	Total Patient	Recovery Group	Non-Recovery Group	*p*-Value
Age	48.20 ± 14.03	43.81 ± 16.56	51.42 ± 10.81	0.1559
Gender, male	148 (49.67%)	64 (50.79%)	84 (48.88%)	0.8287
Recurrent	71 (23.83%)	23 (18.25%)	48 (27.91%)	0.0727
Diabetes mellitus	34 (11.41%)	12 (9.52%)	22 (12.79%)	0.4890
Hypertension	65 (21.81%)	17 (13.49%)	48 (27.91%)	0.0046
Smoke type				0.3746
Non-smoker	224 (75.17%)	91 (72.22%)	133 (77.33%)	
Smoker	60 (20.13%)	30 (23.81%)	30 (17.44%)	
Ex-smoker	14 (4.70%)	5 (3.97%)	9 (5.23%)	
Smoke pack-years	4.58 ± 12.03	5.24 ± 14.25	4.09 ± 10.13	0.5370
Tympanic membrane condition				0.6181
Less than 25%	175 (58.72%)	76 (60.32%)	99 (57.56%)	
25% to 50%	47 (15.77%)	21 (16.67%)	23 (13.37%)	
50% to 75%	38 (12.75%)	15 (11.90%)	23 (13.37%)	
75% over or tube inserted	38 (12.75)	14 (11.11%)	24 (13.95%)	
Perforation margin TSP	66 (22.15%)	28 (22.22%)	38 (22.09%)	1.0
Retraction	84 (28.19%)	28 (22.22%)	56 (32.56%)	0.0674
Attic destruction	159 (53.36%)	73 (57.94%)	86 (50%)	0.2153
Preop otorrhea	96 (32.21%)	46 (36.51%)	50 (29.07%)	0.2180
Preop culture				0.6427
None	164 (55.03%)	65 (51.59%)	99 (57.56%)	
No bacteria/normal flora	62 (20.81%)	30 (23.81%)	32 (18.6%)	
MRSA, CRPA	11 (3.69%)	4 (3.17%)	7 (4.07%)	
Others	61 (20.47%)	27 (21.43%)	34 (19.77%)	
Intraoperative culture				0.4801
None	27 (9.06%)	15 (11.90%)	12 (6.98%)	
No bacteria/normal flora	231 (77.52%)	93 (73.81%)	138 (80.23%)	
MRSA, CRPA	7 (2.35%)	2 (1.59%)	4 (2.33%)	
Others	33 (11.07%)	15 (11.90%)	18 (10.47%)	
Intraoperative eustachian tube findings				0.0000
None	4 (9.06%)	3 (2.38%)	1 (0.58%)	
Patent	174 (77.52%)	90 (71.43%)	84 (48.84%)	
Partially obstructive	31 (2.35%)	10 (7.94%)	21 (12.21%)	
Completely obstructive	89 (11.07%)	23 (18.25%)	66 (38.37%)	
Stapes fixation				0.7280
Unknown	20 (6.23%)	6 (5.26%)	11 (5.85%)	
No	283 (88.18%)	120 (90.23%)	163 (86.70%)	
Yes	18 (6.23%)	7 (5.26%)	11 (7.45%)	
Malleus				0.0195
Intact	50 (16.78%)	22 (19.84%)	25 (14.53%)	
Partial removal/defected	111 (37.25%)	55 (43.65%)	56 (2.91%)	
Total removal/defected	137 (45.97%)	46 (36.51%)	91 (52.91%)	
Tympanoplasty technique				0.3378
None	92 (30.87%)	37 (29.37%)	55 (31.98%)	
Underlay	49 (16.44%)	18 (14.29%)	31 (18.02%)	
Overunderlay	30 (10.07%)	14 (11.11%)	16 (9.30%)	
Overlay	119 (39.93%)	51 (40.48%)	68 (39.53%)	
Umbo-anchoring	8 (2.68%)	6 (4.76%)	2 (1.16%)	
Preoperative AC PTA, dB	55 ± 23.99	45.43 ± 20.47	62.01 ± 24.01	0.2139
Preoperative BC PTA, dB	26.08 ± 17.80	24.96 ± 14.29	31.82 ± 11.44	0.0195
Preoperative ABG, dB	28.92 ± 13.14	31.82 ± 11.44	22.96 ± 14.29	0.0002

Chi-squared test was performed. The data were collected from Korea University Ansan Hospital. MRSA, methicillin-resistant Staphylococcus aureus; CRPA, carbapenem-resistant Pseudomonas aeruginosa; AC PTA, air-conduction pure-tone average; BC PTA, bone-conduction pure-tone average; ABG, air–bone gap; TSP, tympanosclerotic plaque.

**Table 2:** Parameter of hearing recovery.

Parameter	Description
Air-conduction PTA (AC PTA)	The mean of the frequencies at 500 Hz, 1 kHz, 2 kHz, and 4 kHz.
Bone-conduction PTA (BC PTA)	The mean of the frequencies at 500 Hz, 1 kHz, 2 kHz, and 4 kHz.
Air–bone gap (ABG)	The difference between AC PTA and BC PTA.

**Table 3:** Analysis results of the performance of each algorithm.

Algorithm	PPV	Sensitivity	F1 Score
Logistic regression	0.6322 [0.5795–0.6849]	0.6917 [0.5786–0.8047]	0.6528 [0.5841–0.7215]
Decision tree	0.6218 [0.5761–0.6674]	0.7574 [0.6385–0.8743]	0.6751 [0.6130–0.7373]
Random forest	0.5925 [0.5149–0.6702]	0.5686 [0.4255–0.7116]	0.5535 [0.4699–0.6372]
Support vector machine	0.6238 [0.5565–0.6912]	0.5788 [0.4842–0.6734]	0.5917 [0.5321–0.6512]
Light GBM	0.6945 [0.6018–0.7872]	0.5788 [0.4852–0.6725]	0.6204 [0.5512–0.6895]
XGBoost	0.6375 [0.5880–0.6870]	0.5397 [0.4394–0.6401]	0.5777 [0.5028–0.6526]

PPV: positive predictive value.

**Table 4:** Analysis results of the performance of each algorithm of each FPR.

Algorithm	PPV	Sensitivity	F1 Score
With a threshold of 10% FPR			
Logistic regression	0.6518 [0.5629–0.7408]	0.3494 [0.2351–0.4636]	0.4464 [0.3316–0.5612]
Decision tree	0.6939 [0.6244–0.7635]	0.3355 [0.2387–0.4322]	0.4421 [0.3456–0.5385]
Random forest	0.7100 [0.6053–0.8147]	0.3571 [0.2572–0.4569]	0.4678 [0.3582–0.5774]
Support vector machine	0.6689 [0.5487–0.7892]	0.3276 [0.2294–0.4257]	0.4314 [0.3162–0.5466]
Light GBM	0.6179 [0.4549–0.7808]	0.2763 [0.1467–0.4059]	0.3693 [0.2265–0.5121]
XGBoost	0.6161 [0.5409–0.6913]	0.2519 [0.1346–0.3692]	0.3378 [0.2140–0.4617]
With a threshold of 20% FPR			
Logistic regression	0.6577 [0.6052–0.7103]	0.4667 [0.3698–0.5636]	0.5390 [0.4645–0.6134]
Decision tree	0.6512 [0.5947–0.7077]	0.4596 [0.3365–0.5828]	0.5183 [0.4252–0.6115]
Random forest	0.6264 [0.5428–0.7100]	0.4859 [0.3486–0.6232]	0.5375 [0.4199–0.6552]
Support vector machine	0.6343 [0.5254–0.7432]	0.4308 [0.2783–0.5833]	0.4933 [0.3497–0.6368]
Light GBM	0.6646 [0.5920–0.7372]	0.4699 [0.3596–0.5802]	0.5436 [0.4447–0.6424]
XGBoost	0.6117 [0.5725–0.6509]	0.4519 [0.3606–0.5433]	0.5132 [0.4422–0.5842]
With a threshold of 30% FPR			
Logistic regression	0.6195 [0.5525–0.6865]	0.6404 [0.5465–0.7343]	0.6252 [0.5551–0.6953]
Decision tree	0.6214 [0.5777–0.6651]	0.7083 [0.6053–0.8113]	0.6574 [0.5979–0.7168]
Random forest	0.5774 [0.5223–0.6325]	0.6276 [0.5281–0.7270]	0.5995 [0.5238–0.6752]
Support vector machine	0.5255 [0.4491–0.6019]	0.5571 [0.4239–0.6902]	0.5355 [0.4313–0.6397]
Light GBM	0.6158 [0.5390–0.6926]	0.6212 [0.5221–0.7202]	0.6127 [0.5370–0.6884]
XGBoost	0.6291 [0.5971–0.6611]	0.5788 [0.4811–0.6766]	0.5975 [0.5321–0.6630]
With a threshold of 40% FPR			
Logistic regression	0.5452 [0.4893–0.6012]	0.6968 [0.6025–0.7911]	0.6089 [0.5411–0.6767]
Decision tree	0.6194 [0.5746–0.6641]	0.7641 [0.6498–0.8784]	0.6778 [0.6162–0.7394]
Random forest	0.5612 [0.5083–0.6140]	0.6994 [0.5701–0.8286]	0.6188 [0.5331–0.7046]
Support vector machine	0.5450 [0.5053–0.5848]	0.6737 [0.5677–0.7798]	0.5999 [0.5331–0.6668]
Light GBM	0.5480 [0.5057–0.5903]	0.7160 [0.6144–0.8177]	0.6168 [0.5571–0.6766]
XGBoost	0.5944 [0.5550–0.6338]	0.7019 [0.5762–0.8276]	0.6318 [0.5665–0.6971]

PPV: positive predictive value.

## Data Availability

The datasets generated and/or analyzed in the current study are available from the corresponding author upon reasonable request. Code Availability: https://github.com/imdatalab/CWD_recovery (accessed on 6 march 2024).
